# Beijing Friendship Hospital Osteoporosis Self-Assessment Tool for Elderly Male (BFH-OSTM) *vs* Fracture Risk Assessment Tool (FRAX) for identifying painful new osteoporotic vertebral fractures in older Chinese men: a cross-sectional study

**DOI:** 10.1186/s12891-021-04476-2

**Published:** 2021-06-28

**Authors:** Ning An, Ji Sheng Lin, Qi Fei

**Affiliations:** Department of Orthopedics, Beijing Friendship Hospital, Capital Medical University, Beijing, 100050 China

**Keywords:** Osteoporosis, Male, Bone mineral density, Osteoporosis Self-Assessment Tool for Asians, Fracture risk assessment tool, Beijing Friendship Hospital Osteoporosis Self-Assessment Tool for Elderly Male

## Abstract

**Background:**

To compare the validation of four tools for identifying painful new osteoporotic vertebral compression fractures (PNOVCFs) in older Chinese men: bone mineral density (BMD), Asian osteoporosis self-assessment tool (OSTA), World Health Organization fracture risk assessment tool (FRAX) (without BMD) and Beijing Friendship Hospital Osteoporosis Self-Assessment Tool (BFH-OSTM).

**Methods:**

A cross sectional study was conducted from 2013 to 2019. A total of 846 men aged ≥50 were included and were divided into two groups: Fracture Group (patients with PNOVCFs underwent percutaneous vertebroplasty surgery) and Non-Fracture Group (community dwelled subjects for healthy examination). All subjects accepted a dual-energy X-ray BMD test and a structured questionnaire. The results of BMD, OSTA, FRAX and BFH-OSTM scores were assessed and receiver-operating characteristic (ROC) curves were generated to compare the validity of four tools for identifying PNOVCFs. Optimal cutoff points, sensitivity, specificity, and areas under the ROC curves (AUCs) were determined.

**Results:**

There were significant differences including BMD T score (femoral neck, total hip and L1-L4), OSTA, FRAX and BFH-OSTM scores between Fracture group and Non-fracture group. Compared to BMD and OSTA, BFH-OSTM and FRAX had better predictive value, the sensitivity, specificity and AUC value are 0.841, 81.29%, 70.67% and 0.796, 74.85%, 78.52%, respectively. Compared with FRAX, the BFH-OSTM has a better AUC value.

**Conclusions:**

Both BFH-OSTM and FRAX can be used to identify POVCFs, However, BFH-OSTM model may be a more simple and effective tool to identify the risk of POVCFs in Chinese elderly men.

## Introduction

Osteoporotic vertebral compression fracture (OVCF) is the most common complication of primary osteoporosis, which often occurs in postmenopausal women, and also troubles many elderly men. 1/5 of men around the world are threatened by osteoporotic fractures after the age of 50 [1]. Fragility fractures can cause substantial pain and severe disability, often leading to a reduced quality of life, and vertebral fractures are associated with decreased life expectancy [2]. Osteoporotic vertebral fractures accounted for 0.83% of the global burden of non-communicable diseases [3]. More than 40% of patients failed to achieve significant pain relief within 12 months [4, 5]. More worryingly, the long-term mortality rate of patients with a history of OVCF was significantly higher than that of the general population. Finally, the hospital mortality rate of OVCF patients ranged from 0.3% to 1.7%. This also invisibly increases the social and economic burden.

How to early identify the painful new osteoporotic vertebral compression fractures (PNOVCFs) is facing major challenges all over the world, especially in primary hospitals [6]. The clinical onset of Older men PNOVCF is hidden, the patients have only a history of mild low energy injury or even no any trauma history, the pain degree of the patients varies greatly, some of them develop into chronic pain, and the physical examination often does not have clear localization signs (some patients even complain about the pain site is not consistent with the actual fracture level) [7]. These characteristics make PNOVCF easy to be misdirected or missed, especially in primary hospitals with limited professional experience and equipment. New vertebral fracture fractures cause unbearable pain and a series of complications caused by bed rest are extremely painful for patients. Early screening and diagnosis of high-risk male may play an important role in reducing the incidence of severe events and mortality. Therefore, it is very necessary to develop an appropriate simple screening tool based on clinical risk factors for PNOVCF, especially to aid physicians with limited professional experience and equipment [8].

OSTA, an Asian osteoporosis self-assessment tool developed by Koh et al, is based on age and weight to assess the risk of osteoporosis with high sensitivity and acceptable specificity [9]. Some clinical results suggest that OSTA (cutoff < −1) revealed a sensitivity of 32.3%, a specificity of 92.3%, and AUC of 0.618 in identifying subjects with osteoporotic vertebral compression fracture in population aged 40 years and above residing in Malaysia [10]. Among them, male accounts for nearly 50%, which partly show the identifying effect of OSTA on older men OVCF. Our previous results also showed that the AUC was 0.661 with a cutoff of −1.2 and sensitivity of 53.15% and, a specificity of 76.88% in the population of Chinese men aged 50 years and above consecutively recruited from the Osteoporosis Clinic at Beijing Friendship Hospital [11]. It may also be helpful to identify postmenopausal women with OVCF. However, OSTA still lacks sufficient confirmation in identifying PNOVCF.

In 2008, the World Health Organization introduced the fracture risk assessment tool FRAX to assess the absolute risk of osteoporotic fractures in patients [12]. To predict the likelihood of severe osteoporotic fractures within 10 years, FRAX considered the interaction of risk factors such as age, gender, and personal and family history. In addition, the incidence of fractures varies widely from country to country, and FRAX can calibrate risk factors according to different countries. Our preliminary cross-sectional study confirmed that FRAX can be used as a predictive tool to help detect PNOVCF. The AUC of the FRAX tool was 0.738 with a cutoff of 2.9%, a sensitivity of 81.98% and a specificity of 62.0% [11]. However, in clinical practice, FRAX needs to include seven risk factors, which is not easy to be promoted in primary or community hospitals.

Fragile fracture history is an important risk factor not only for osteoporosis, but also for OVCF [13, 14]. However, our previous study developed a clinical screening tool (BFH-OSTM) based on two clinical risk factors including the history of fragility fracture. Previous studies have confirmed that it can well identify male osteoporosis, the BFH-OSTM index (cutoff = 70) had a sensitivity of 85% and specificity of 53% for identifying osteoporosis according to the WHO criteria, with an area under the ROC curve of 0.763. However, is difficult to know whether it has any value in detecting and identifying PNOVCF [15].

Therefore, this cross sectional study evaluated and compared the validation of BMD, OSTA and FRAX (without BMD) and our constructed BFH-OSTM in identifying POVCF in a Chinese elderly men population.

## Materials and methods

**Fig. 1:**
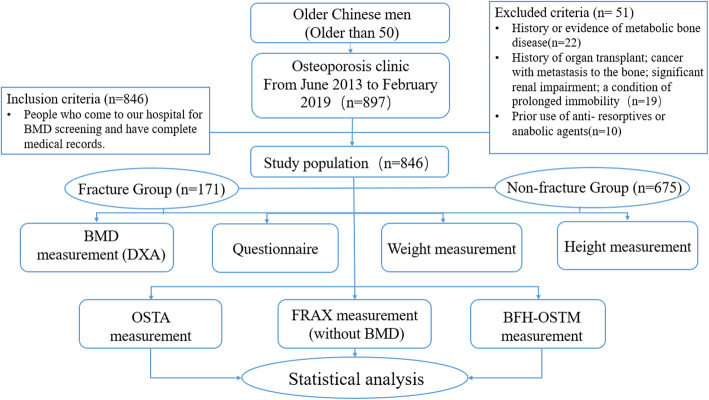
*BMD* bone mineral density, *OSTA* Osteoporosis self-Assessment Tool for Asians, *FRAX* fracture risk assessment tool, *BFH-OSTM* Beijing Friendship Hospital Osteoporosis Self-Assessment Tool for Elderly Male

This cross sectional study was approved by the Ethics Committee of Beijing Friendship Hospital, Capital Medical University, and all subjects provided signed informed consent. Our study confirms that all methods are implemented in accordance with the relevant guidelines and regulations. The main flow chart of the study was shown in Fig. 1.

### Study population

The subjects in this study included Chinese men aged ≥50 years who came to the Orthopedic Clinic of Beijing Friendship Hospital from June 2013 to February 2019. All subjects accepted a dual-energy X-ray BMD test and a structured questionnaire. These men included confirmed POVCFs patients with clinical symptoms verified by X-ray, MRI and other examinations within the past 6 months (Fracture group); and community dwelled men for health examination and bone mineral density screening (Non-fracture group). All subjects were required to fill in a questionnaire by a trained interviewer to provide information regarding demographic variables and clinical risk factors for osteoporosis using a structured table. We set these potential risk factors in the questionnaire were identified from previous researches. These factors included age, height, weight, body mass index (BMI), previous fracture, current smoking, consumption of three of more alcoholic drinks per day, glucocorticoids, rheumatoid arthritis and parent fractured hip history [16]. Measure height with a stadiometer (Mahr GmbH, Gottingen, Germany). The weight was measured on an electronic scale (Tanita, Tokyo, Japan), and the subjects wore lightweight indoor clothes and without shoes.

Men in the Fracture group and the Non-fracture group both met the following inclusion criteria: aged ≥50 years; Han Chinese nationality; lived locally ≥20 years; willing to participate in this study and signed the informed consent.

Excluded was anyone with a history or evidence of metabolic bone disease (such as type I diabetes, parathyroid dysfunction, Paget’s disease, osteomalacia); the history of organ transplant; bone metastasis of cancer; severe renal impairment; a condition of prolonged immobility (such as spinal cord injury, stroke, muscular dystrophy or ankylosis spondylitis); or the previous use of anti-resorptive drugs (eg, bisphosphonate, estrogen, selective estrogen receptor modulators, and calcitonin) or anabolic agents (eg, fluoride or parathyroid hormone) will be included [17]. The main indications for PVP for the surgery include the following: (1) Acute OVCF (Magnetic resonance imaging [MRI] on T1-weighted images showed a low signal. MRI on T2-weighted images and short tau inversion recovery sequences showed high signal; (2) VAS (visual analogue scale) ≥6; (3) The patient refused to receive conservative treatment [18].

### Fracture group and identification of POVCF

We defined four necessary clinical criteria to determine POVCF. These standards are as following [11]: (1) Men ≥50 years old with no obvious history of trauma or fracture history of low energy trauma. A low-energy traumatic fracture is defined as a fracture caused by a fall from a standing or lower position. (2) There were clinical symptoms such as low back pain within 6 months before the bone mineral density scans. (3) We evaluated the clinical signs of osteoporotic vertebral fracture by X-ray and MRI. On the X-ray film, the signs include the reduction of the height of the anterior, middle and posterior dimensions of the vertebral body >20% of the vertebral body’s area in a lateral-view image of the thoracic/lumbar spine; Or there are endplate deformities, lack of parallelism, and general changes in appearance relative to adjacent vertebrae. On MRI images, the signs of osteoporotic vertebral compression fractures included newly found bone marrow edema on sagittal T1-weighted images and fat-suppressed T2-weighted images. (4) There is no history or evidence of metabolic bone disease or cancer [19].

### BFH-OSTM

In this model [15], only weight and history of the previous fracture were selected in the ultimate model. The new model had been named the Beijing Friendship Hospital Osteoporosis Self-Assessment Tool for Elderly Male (BFH-OSTM) [20]. The model was calculated using the following formula:

$$ \left(\mathrm{Body}\ \mathrm{weight},\mathrm{kg}-\mathrm{history}\ \mathrm{of}\ \mathrm{previous}\ \mathrm{fragility}\ \mathrm{fracture}\ \left[\mathrm{no}=0,\mathrm{yes}=1\right]\times 7\right). $$

Two key factors of our model were collected as follows: medical records were reviewed to record body weight and previous fragility history information at admission and information on the questionnaire was collected in the fracture group and the non-fracture group. We defined a fragility fracture as men ≥50 years old with no obvious history of trauma or fracture history of low energy trauma. A low-energy traumatic fracture is defined as a fracture caused by a fall from a standing or lower position. Fragility fractures occur most commonly in the spine (vertebrae), hip (proximal femur) and wrist (distal radius). They may also occur in the arm (humerus), pelvis, ribs and other bones. Fragility fractures are defined as fractures associated with low BMD and include clinical spine, forearm, hip and shoulder fractures. For example, a man whose body weight was 65 kg with a previous fragility fracture would have an index of: 65 − 1 × 7 = 58.

### BMD measurements

All the enrolled men accepted the BMD measurement of the hip and spine by DXA at our Hospital.

We use the Wi densitometer (Hologic Inc., Bedford, MA, USA) to measure the BMD of the left lumbar vertebrae (L1~L4), the left femur and total hip. In order to standardize the measurement, quality control is carried out every day before the initial measurement. These men had short-term repeatability values of less than 1% at the lumbar spine, femoral neck and total hip [21]. Throughout the study, all DXA scans were performed by the same experienced and qualified technical expert. The BMD T score was calculated automatically by the system. T score refers to the average bone mineral density of young Chinese men: L1~L4, 1.017 ± 0.117 g/cm^2^, femoral neck 0.909 ± 0.116 g/cm^2^, total hip 0.993 ± 0.121 g/cm^2^.

### OSTA score

The osteoporosis self-assessment tool for Asians (OSTA) was first developed by Koh et al. in 2001 [9]. In the final formula, only age and body weight are selected as influencing factors, and the formula is as follows [10]:

$$ \mathrm{OSTA}\ \mathrm{score}=\left(\mathrm{body}\ \mathrm{weight},\mathrm{kg}-\mathrm{age},\mathrm{years}\right)\times 0.2. $$

Then only the integer part is taken as the result of the calculation. For example, a 70-year – old man whose body weight was 75 kg would have an index of: (75 − 70) × 0.2 = 1.

### FRAX score

FRAX is a computer algorithm based on the use of clinical risk factors (http://www.sheffield.ac.uk/FRAX) and is also the most widely validated and widely used tool for male and female fracture risk assessment [22]. It combines the risk of fracture and the risk of death, and constructs four models to calculate the probability of fracture. The 10-year fracture probability can not only be obtained by clinical risk factors, but also can be more accurately predicted by combining with the bone mineral density of the femoral neck. As the probability of fracture also varies significantly in different regions of the world, the FRAX model is calibrated and modified according to the epidemiological data of fracture and death in diverse regions. We chose the version of FRAX in the Chinese mainland. Because we focused on the ability of FRAX tools to identify and predict painful osteoporotic vertebral compression fractures in this study, we selected a model for the likelihood of osteoporotic fractures within 10 years without bone mineral density measurements [23]. In this study, we use the Major osteoporotic data of FRAX (without BMD).

### Statistical analysis

In this study, the descriptive statistics of demographic and baseline characteristics are expressed as the average ± standard deviation of continuous variables or the percentage of classified variables. The measured data are described as mean ± standard deviation in the normal distribution, otherwise described as median and quartile range. A Chi-square test was used to compare the counting data.

The differences of BMD, OSTA, FRAX score and BFH-OSTM between the Fracture group and the non-Fracture group were tested by t-test for two independent samples (if it was normal distribution and uniform variance), while the non-normal distribution was tested by a non-parametric test. The effectiveness of four tools for identifying OVCF was evaluated by receiver-operating characteristic (ROC) curve analysis, which compared sensitivity to (1-specificity). The predicted values of the (AUC) determination tool based on the area under the ROC curve are as follows [24]: AUC < 0.5; less predictive, 0.5 < AUC < 0.7; moderately predictive, 0.7 < AUC < 0.9; highly predictive, 0.9 < AUC < 1; and perfectly predictive, AUC = 1. Build the ROC curve and estimate AUC and its 95% confidence interval (CI) using SPSS version 26.0 and MedCalc version 11.5.0.0. The *p* value <0.05 was considered statistically significant.

## Results

A sample of 897 men aged 50 initially enrolled in the study. According to the inclusion and exclusion criteria, a total of 51 subjects were excluded from the study, so 846 subjects were analyzed (Table 1). These included 171 men who suffered POVCF within 6 months before the BMD measurement (Fracture group) and 675 healthy community-based men (Non-fracture group).

**Table 1:** Summary of descriptive characteristics of Fracture Group and Non-Fracture Group

Characteristics	Fracture Group	Non-Fracture Group	*p* (t/χ^2^)
Subjects, n	171	675	
Weight, kg	68.00 (60.00, 72.00)	75.00 (68.00, 80.00)	<0.001 (11.776)
Height, cm	170.00 (165.00, 173.00)	170.00 (167.00, 174.00)	0.041 (2.056)
BMI, kg/m^2^	23.05 (21.22, 24.69)	25.65 (23.84, 27.40)	<0.001 (11.874)
Previous fracture	94/171 (55%)	34/675 (5%)	<0.001 (264.925)
BMD, g/cm^2^			
Femoral neck	0.69 (0.59, 0.76)	0.81 (0.73, 0.90)	<0.001 (12.127)
Total hip	0.81 (0.70, 0.89)	0.93 (0.84, 1.02)	<0.001 (10.068)
L1-L4	0.83 (0.73, 0.92)	0.99 (0.89, 1.10)	<0.001 (12.676)
Family history	2/171 (1.2%)	43/675 (6.4%)	0.007 (7.328)
Current smoker	42 (24.6%)	356 (52.7%)	<0.001 (43.488)
Alcohol 30 g/d	29 (17%)	333 (49.3%)	<0.001 (58.414)

**Notes**: Data are presented as n (%) or mean ± standard deviation

Between the Fracture group and the non-Fracture group, there were considerable differences in weight, height, previous fracture, family history and BMDs of the femoral neck, total hip and L1~L4 [25, 26]. In particular, the body mass index was highest in the Non-fracture group relative to the Fracture group. Men in the Fracture group experienced more fractures and had a lower average BMDs than men in the control group.

Compared to the control group, the BMI of the Fracture group range from 14.17 kg/m^2^ to 31.6 kg/m^2^, and that of the non-Fracture group was between 17.72 kg/m^2^ and 37.18 kg/m^2^. Previous fractures accounted for 20.21% of the total sample (n = 171), and 5.32% of the subjects had a family history of osteoporosis (n = 45). At present, smokers are accountable for 47.04% of the study population (n = 398), and 42.79% of people drink more than 30 g of alcohol per day (n = 362) [22]. There were significant differences in weight, height, BMI, previous fracture, BMD, family history, current smokers and alcohol over 30 g/d between the fracture group and the non-fracture group (*p* < 0.05).

### BMD T-scores and OXTA and FRAX indices and BFH-OSTM

There were significant differences in BMD T-score, FRAX, OSTA and BFH-OSTM scores between the Fracture group and the non-Fracture group (Table 2). The BMD scores of total hip, femoral neck and L1~L4, and the BFH-OSTM score, and OSTA score in the Fracture group were significantly lower than those in the non-Fracture group, while the FRAX index was significantly higher than that in the non-Fracture group.

**Table 2:** BMD T-score, OSTA, FRAX and BFHOSTM scores of Fracture Group and Non-Fracture Group

Parameter	Fracture Group	Non-Fracture Group	z/t	*p*-value
Subjects, n	171	675		
BMD, T-score, mean ± SD				
Femoral neck	−1.80 (−2.70, −1.30)	−0.90 (−1.50, −0.20)	−12.00	<0.001
Total hip	−1.70 (−2.70, −1.10)	−0.70 (−1.30, −0.10)	−11.05	<0.001
L1-L4	−1.60 (−2.4, −0.80)	−0.90 (−1.8, 0.10)	−6.86	<0.001
OSTA, mean ± SD	−1.20 (−3.8, 1.40)	2.00 (0.20, 4.00)	−10.72	<0.001
FRAX (%)	3.50 (2.90, 4.70)	2.40 (2.00, 2.80)	−12.00	<0.001
BFH-OSTM (%)	63 (56, 68)	74 (68, 80)	−13.80	<0.001

**Abbreviations**: *BMD* bone mineral density, *OSTA* Osteoporosis self-Assessment Tool for Asians, *FRAX* fracture risk assessment tool, *BFH-OSTM* Beijing Friendship Hospital Osteoporosis Self-Assessment Tool for Elderly Male

### BMD T-scores

**Fig. 2:**
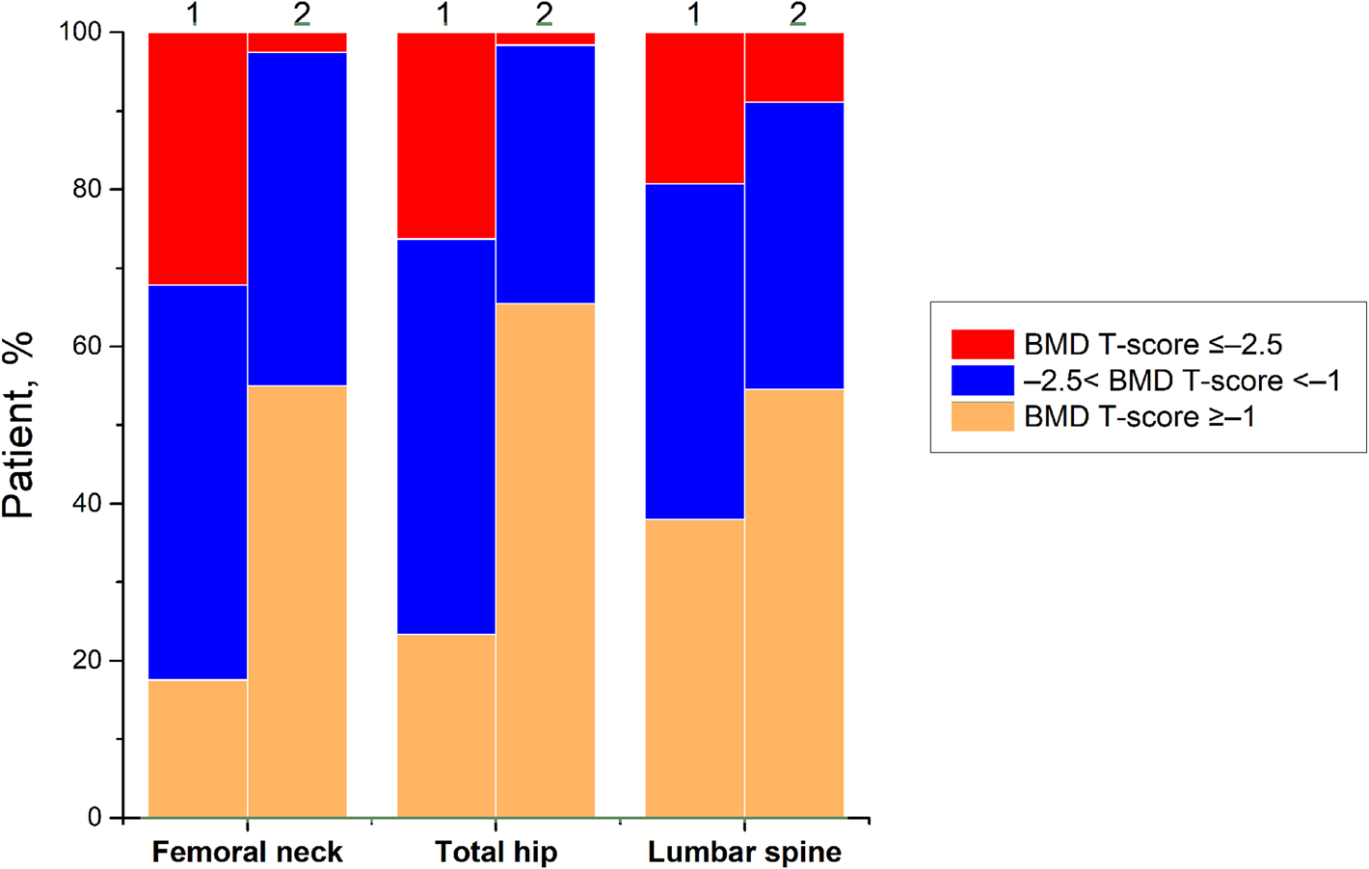
Proportions of BMD T-scores at different sites in the Fracture (1) and Non-Fracture (2) groups

For men in the Fracture group, only 50.9% were found to have osteoporosis (WHO criteria) which has BMD T-scores below −2.5 at the femoral neck, or total hip, or lumbar spine (Fig. 2). For the non-Fracture group, these percentages were only 2.5%, 1.6%, and 8.9%, respectively. The AUC of the BMD for estimating the risk of OVCF at the femoral neck, hip, and lumbar spine were 0.779, 0.776 and 0.650 with optimal cutoffs of −1.4, −1.4 and −0.7.

### Evaluation and comparison of BMD T-score, OSTA, FRAX and BFH-OSTM

**Fig. 3:**
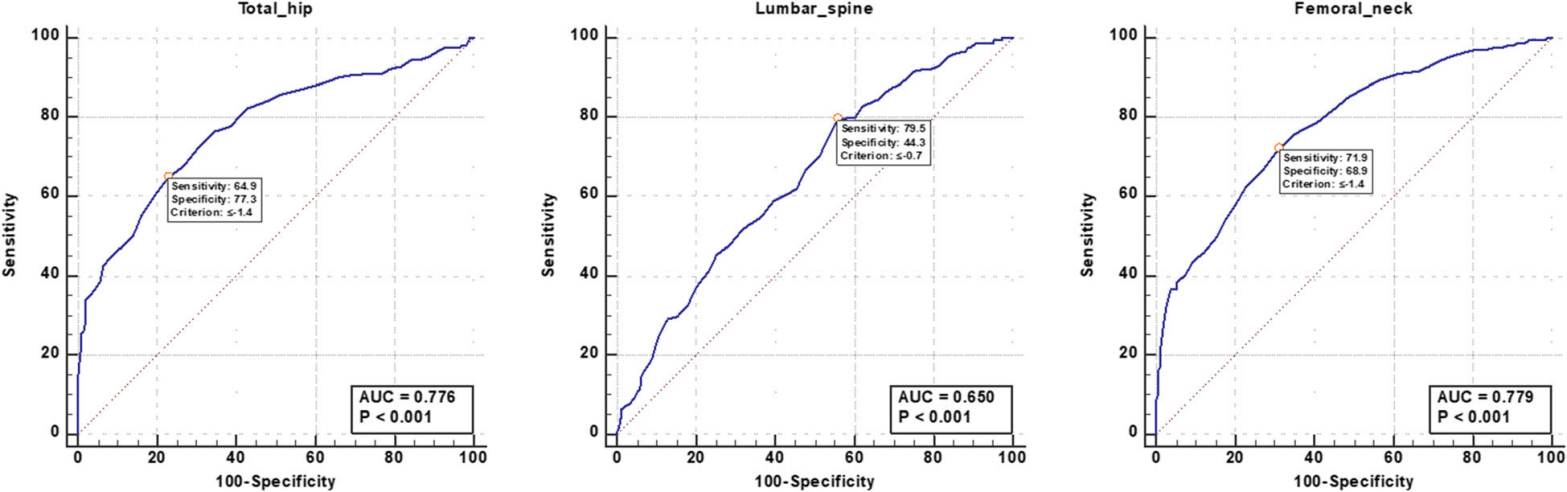
ROC curve of the BMD measurement at different sites for identifying PNOVCF with optimal cutoff value. *BMD* bone mineral density, *OSTA* Osteoporosis self-Assessment Tool for Asians, *FRAX* fracture risk assessment tool, *BFHOSTM* Beijing Friendship Hospital Osteoporosis Self-Assessment Tool for Elderly Male

**Fig. 4:**
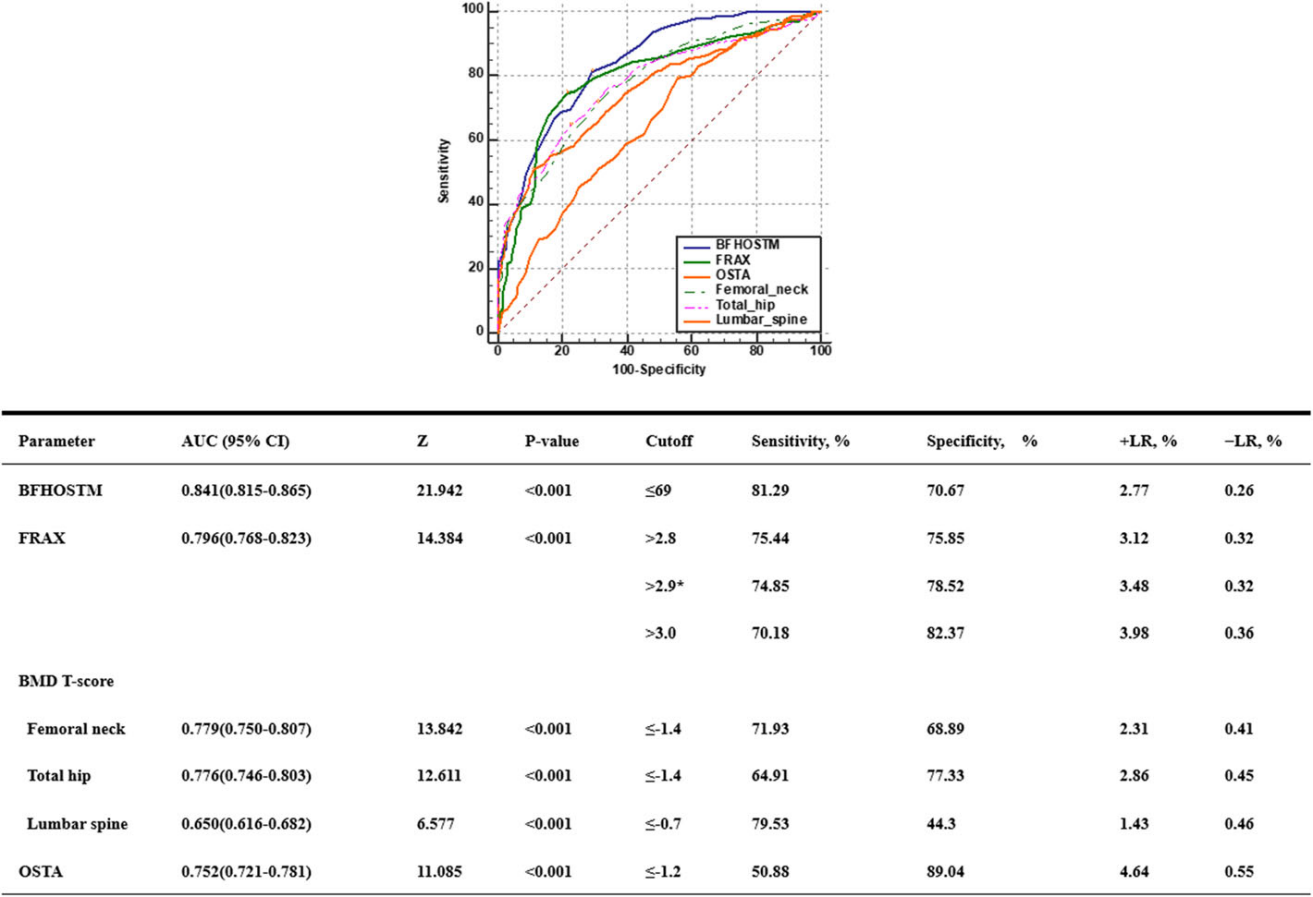
AUC, sensitivity and specificity values of the FRAX, BMD T-score, OSTA and BFH-OSTM for identifying PNOVCF. Comparison of different AUCs (BMD T-score, OSTA, FRAX and BFHOSTM for identifying OVCF). *Optimal FRAX cutoff; +LR: positive likelihood ratio; −LR: negative likelihood ratio. *BMD* bone mineral density, *OSTA* Osteoporosis self-Assessment Tool for Asians, *FRAX* fracture risk assessment tool, *BFH-OSTM* Beijing Friendship Hospital Osteoporosis Self-Assessment Tool for Elderly Male, *AUC* area under the receiver operating characteristic curve, *OVCF* osteoporotic vertebral compression fracture

**Fig. 5:**
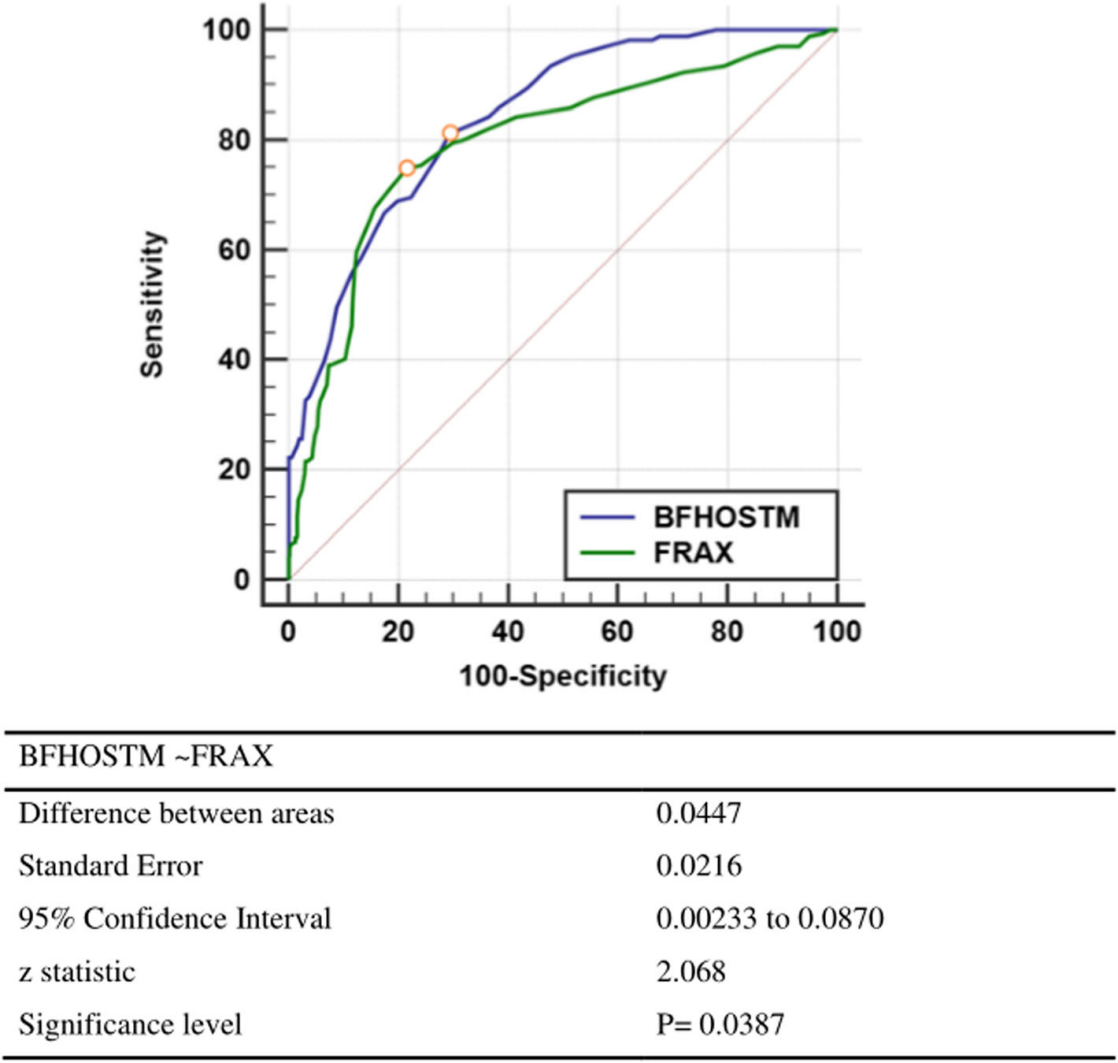
Comparison of different AUCs (FRAX and BFHOSTM for identifying OVCF)

First of all, our BFH-OSTM model performs better in it, with an AUC value of 0.841 (95%CI: 0.815–0.865 Z = 21.942, *p* < 0.001), with a cutoff of 69, and a sensitivity and specificity of 81.29% and 70.67%. The AUC value of the FRAX tool (without BMD) is 0.796 (95%CI: 0.768–0.823 Z = 14.384, *p* < 0.001), the cutoff value is 2.9%, and the sensitivity and specificity are 74.85% and 78.52%, respectively. Compared with FRAX, BFH-OSTM model may be a more effective tool to determine the risk of PNOVCF in this elderly Chinese men. The Z-value between BFH-OSTM and FRAX are 2.068 and the *p*-value are 0.0387 < 0.05, so the difference is statistically significant (Fig. 5). The AUC values of BMD at femoral neck, hip and lumbar vertebrae for the diagnosis of OVCF were 0.779 (95%CI: 0.750–0.807, Z = 13.842, *p* < 0.001), 0.776 (95%CI: 0.746–0.803, Z = 12.611, *p* < 0.001) and 0.650 (95%CI: 0.616–0.682, Z = 6.577, *p* < 0.001), respectively. The cutoff values are −1.4, −1.4 and −0.7. For the OSTA model, the AUC value was 0.752 (95%CI: 0.721–0.781, Z = 11.085%, *p* < 0.001), and when the cutoff value was −1.2, the sensitivity and specificity were 50.88% and 89.04%. (Figs. 3 and 4)

## Discussion

This cross sectional study compared the validity of BMD/OSTA/FRAX (without BMD) and BFH-OSTM for identifying POVCF in Chinese men aged 50 and over. According to diagnostic criteria of POVCF issued by our criteria, these tools are suitable for comparison between healthy men and patients with PNOVCF.

Our study showed that for BMD, the AUC values for assessing the risk of PNOVCF of the femoral neck, hip and lumbar vertebral were 0.779, 0.776 and 0.650, respectively, and the corresponding optimal critical values were −1.4, −1.4 and −0.7. This indicates that BMD measurements of the femoral neck, hip and lumbar vertebral are only moderately predictive. Among the three parts, the identifiability of the lumbar spine was higher, and its sensitivity was 79.53%. As a screening method, the specificity of total hip was 77.33%. However, our results show that the sensitivity of BMD in assessing the risk of fracture is moderate, with a sensitivity of 71.93% in the femoral neck, 64.91% in the total hip and 79.53% in the lumbar spine, respectively, and the specificity in the lumbar spine is only 44.3%, so the specificity is less acceptable in the lumbar spine. Our previous research showed that the AUC for estimating the risk of fracture at the femoral neck, hip, and lumbar spine were 0.706, 0.711, and 0.706, respectively, with optimal cutoffs of −2.5, −1.4, and −1.6, with had a sensitivity of 42.34%, 67.57%, 52.25% and specificity of 89.87%, 65.45%, 77.14%. Due to the high cost of central dual-energy X-ray absorptiometry, BMD is not suitable as a preliminary screening tool in primary hospitals. In the fracture group, 17.5%, 23.4% and 38.0% of the patients had normal BMD at the three parts of femoral neck, total hip and lumbar spine respectively, so the value of bone mineral density as a predictor of PNOVCF was limited. Therefore, we urgently need a screening tool with higher accuracy and simplicity than bone mineral density measurement to identify PNOVCF. Because there is no such equipment in primary hospitals, this tool should not be used as a screening tool.

As showed in Table 1, the average weight, height and BMI of the Fracture group were lower than those of the non-Fracture group. Patients in the Fracture group experienced more fragility fractures than the non-Fracture group, so we think that low weight and previous fragility fracture history of osteoporosis are also risk factors for PNOVCF. This is consistent with the traditional clinical view [27]. If the height is lower than that of the general healthy people, it may be due to the physiological characteristics of the spine, the vertebral body of the osteoporosis patient is more likely to be compressed, and the morphological changes of the vertebral body and intervertebral space lead to the shortening of the length of the spine [28]. The calculation of OSTA is very simple, based only on the two influencing factors of age and body weight, which is simpler than BMD measurement, and is suitable for the risk assessment of osteoporosis in postmenopausal Asian women. In recently published reports, data show that the OSTA index can also be used to predict the risk of osteoporosis in elderly Chinese men, but the prediction of new osteoporotic vertebral compression fractures in this population has not been confirmed [29]. Our previous and current study shows that there is a significant difference in the distribution of OSTA score between the Fracture group and the non-Fracture group. Its ability to recognize OVCF (AUC = 0.752) is slightly lower than that of the femoral neck and hip, but better than that of the lumbar spine. However, the disadvantage that cannot be ignored is low sensitivity (50.88%). For screening tools, we focus more on high sensitivity than on high specificity, as fewer patients will undergo unnecessary treatment or invasive tests [30]. so the OSTA index may not be applicable to the prediction of PNOVCF in Chinese elderly men, which run counter to the purpose of our screening.

The FRAX algorithm was developed to assess the risk of osteoporotic fractures of the hip, spine, distal forearm and shoulder in 10 years and has been recommended by the World Health Organization. Because it does not need BMD measurement, the patient data collection is more comprehensive, so its ability to distinguish PNOVCF is indeed stronger than BMD measurement and OSTA score [23]. The AUC value of FRAX in the diagnosis of OVCF risk was 0.796, and its sensitivity and specificity were 74.85% and 78.52% respectively at the optimal critical value. Among the tools are tested in the present study, FRAX had a higher discriminating ability for identifying PNOVCF, followed by OSTA and BMD. In clinical practice, FRAX needs to include many risk factors and be equipped with corresponding hardware and software, so it has certain limitations [26, 31].

Our BFH-OSTM is a calculation model based on multiple regression analysis of data from multiple centers, and two risk factors, body weight and previous fragility fracture history are selected. Compared with FRAX, BFH-OSTM model may be an effective tool to determine the risk of PNOVCF in this elderly Chinese men population. The performance value of BFH-OSTA is better than FRAX (*p* < 0.05). The formula is (body weight [kg] − history of previous fracture [no = 0, yes = 1] × 7). BFH-OSTM can not only predict osteoporosis, but also can be used for early detection of PNOVCF, and the value of cutoff may be different. The optimal cutoff value for identifying PNOVCF is 69, and the sensitivity and specificity are 81.29% and 70.67%, respectively, and the area under the curve AUC is 0.841. The ability to identify PNOVCF is significantly improved, and the sensitivity is also higher (Fig. 5). Compared with FRAX, the predictive value of our BFH-OSTM is obviously better than that of FRAX. We think that the cutoff value may be slightly different in different people and regions, which need to be further confirmed.

Among the tools tested in this study, BFH-OSTM had the highest discriminative validity in identifying PNOVCF in the elderly men population, with approximate high identification, followed by FRAX, with better BMD in the femoral neck and hip than OSTA. In this study, compared with OSTA and BMD T scores, FRAX score (without BMD) includes more related risk or protective factors and adapts to local conditions, so it has higher identification value, but its universality also limits its promotion and application. Despite the availability of computer software to simplify the calculation. However, the complexity of data collection also makes clinical evaluation difficult, which is not suitable for large-scale screening and community application. Our BFH-OSTM model is a multi-factor analysis model, which accurately captures the two influencing factors of the most critical body weight and previous brittle fracture history, which is simple enough, but at the same time obtains the best predictive value of PNOVCF and ensures the sensitivity and specificity of screening, so it is easy to popularize and apply [32].

Our research presents several noteworthy advantages. First of all, our research is a cross-sectional study, so the information obtained is not retrospective. Second, due to the rigor of data collection, our study shows that the age and weight of subjects are recorded while measuring bone mineral density, and all diagnoses and results are made by experienced physicians. Third, we imposed strict inclusion and exclusion criteria to exclude the effects of other factors. Finally, our research has important clinical significance; it can help inexperienced doctors in primary hospitals or community health service centers to detect PNOVCF as early as possible. More importantly, there is no need for the learning curve, compared with the traditionally recognized ability of FRAX to assess the risk of fracture, BFH-OSTM is a more simple, direct and effective model for clinicians.

However, current research still has some limitations. First of all, we only collected the subjects recruited from a hospital, which is a single-center study, so we cannot fully represent the entire demographic data of the people's Republic of China. Secondly, we suggest that more centers should participate to improve big data and verify the accuracy of the model at the same time.

## Conclusion

Our study found that neither BMD nor OSTA is sufficient to identify the risk of PNOVCF in clinical practice. Compared to FRAX, BFH-OSTM model may be a more simple and effective tool to determine the risk of PNOVCF in this elderly Chinese men population.

## Data Availability

The datasets used and/or analysed during the current study are available from the corresponding author on reasonable request.
